# Role of DNA methylation in miR-200c/141 cluster silencing in invasive breast cancer cells

**DOI:** 10.1186/1756-0500-3-219

**Published:** 2010-08-03

**Authors:** Rui Neves, Christina Scheel, Sandra Weinhold, Ellen Honisch, Katharina M Iwaniuk, Hans-Ingo Trompeter, Dieter Niederacher, Peter Wernet, Simeon Santourlidis, Markus Uhrberg

**Affiliations:** 1 Institute for Transplantation Diagnostics and Cell Therapeutics, University Clinic Düsseldorf, Moorenstr. 5, Building 14.80, 40225 Düsseldorf, Germany; 2 Whitehead Institute for Biomedical Research, 9 Cambridge Center, Cambridge, Massachusetts 02142, USA; 3 Department of Gynaecology and Obstetrics, University Clinic Düsseldorf, Moorenstr. 5, Building 14.80, 40225 Düsseldorf, Germany

## Abstract

**Background:**

The miR-200c/141 cluster has recently been implicated in the epithelial to mesenchymal transition (EMT) process. The expression of these two miRNAs is inversely correlated with tumorigenicity and invasiveness in several human cancers. The role of these miRNAs in cancer progression is based in part on their capacity to target the EMT activators ZEB1 and ZEB2, two transcription factors, which in turn repress expression of E-cadherin. Little is known about the regulation of the mir200c/141 cluster, whose targeting has been proposed as a promising new therapy for the most aggressive tumors.

**Findings:**

We show that the miR-200c/141 cluster is repressed by DNA methylation of a CpG island located in the promoter region of these miRNAs. Whereas in vitro methylation of the miR-200c/141 promoter led to shutdown of promoter activity, treatment with a demethylating agent caused transcriptional reactivation in breast cancer cells formerly lacking expression of miR-200c and miR-141. More importantly, we observed that DNA methylation of the identified miR-200c/141 promoter was tightly correlated with phenotype and the invasive capacity in a panel of 8 human breast cancer cell lines. In line with this, in vitro induction of EMT by ectopic expression of the EMT transcription factor Twist in human immortalized mammary epithelial cells (HMLE) was accompanied by increased DNA methylation and concomitant repression of the miR-200c/141 locus.

**Conclusions:**

The present study demonstrates that expression of the miR-200c/141 cluster is regulated by DNA methylation, suggesting epigenetic regulation of this miRNA locus in aggressive breast cancer cell lines as well as untransformed mammary epithelial cells. This epigenetic silencing mechanism might represent a novel component of the regulatory circuit for the maintenance of EMT programs in cancer and normal cells.

## Findings

Epithelial to mesenchymal transition (EMT) is considered an essential early step in tumor metastasis formation by controlling the detachment of invasive cancer cells from the primary tumor [1]. Interestingly, EMT is also seen as a facilitator of tissue remodeling during embryonic development. The phenotypical changes and the gain of invasive capacity occurring during EMT are consequences of a cascade of events ultimately leading to downregulation of cell-to-cell adhesion proteins such as E-cadherin. Recently, specific microRNAs (miRNAs), namely members of the miRNA-200 family including miR-200c and miR-141, have been implicated in this process [2-4].

MiRNAs are evolutionary conserved small RNAs, able to modulate gene expression by inhibiting the protein translation process and/or degrading the respective target messenger RNA [5]. They have been shown to participate in many cellular processes including tumorigenesis and specific miRNAs have been assigned either oncogenic or tumor suppressor roles [6]. With respect to the EMT process, observations suggest that members of the miRNA-200 family (especially the two clustered miRNAs miR-200c and miR-141) play a prominent role as metastasis suppressor genes by preventing the expression of zinc finger E-box binding homeobox 1 (ZEB1), which in turn promotes EMT and the switch to an invasive phenotype [4,7-10]. Importantly, loss of expression of miRNA-200 family members correlates with EMT in various tumor entities such as breast [4], renal [11], and ovarian [2] cancer and thus seems to be a conserved pathway promoting metastasis formation.

During tumorigenesis and EMT, also epigenetic mechanisms, in particular DNA methylation, play a decisive role and contribute to the regulation of key factors involved in this process. While hypermethylation is observed in regulatory regions of many tumor suppressor genes leading to their transcriptional silencing (e.g. E-cadherin [12]), on the global level genome-wide DNA demethylation is observed [13].

**Figure 1:**
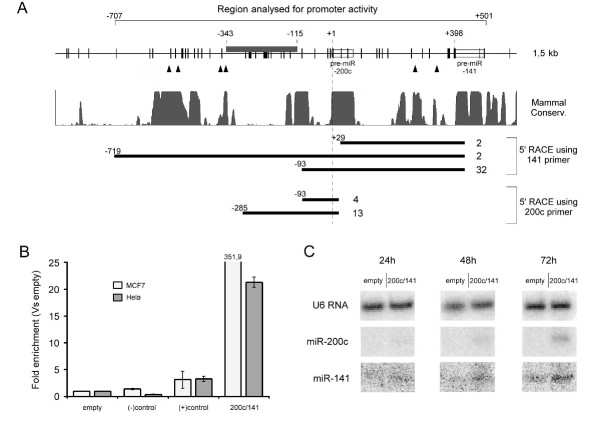
**The promoter of the miR-200c/141 cluster is located in a CpG-rich region**. **(A) **Analysis of the genomic region encompassing the miR-200c/141 cluster. Individual CpG dinucleotides are indicated by short vertical bars, the CpG island between positions -343 and -115 is represented by a dark grey box. Known ZEB1 binding sites [19] are marked with "▲". The degree of conservation among 17 mammalian species is shown in the middle part (area plot). In the lower part, the results of 5' Rapid Amplification of cDNA ends (5' RACE) experiments are depicted. Starting positions of clones representing different transcription start sites are indicated on the left, the number of identified clones indicated on the right side. **(B) **Luciferase reporter gene assays of empty vector (empty) (pMOD vector, Invivogen), a control insert lacking any promoter activity ((-) control) (intronic fraction of KIR3DL2 gene), an insert constituted by the GAPDH promoter ((+) control), and an insert encompassing positions -707 to +501 as indicated in (A). **(C) **Northern blot results using a U6 RNA, a miR-200c, and a miR-141 probe on RNA extracted from Hela cells transfected with pMOD empty vector or the pMOD vector containing the miR-200c/141 genomic region. Bands shown for the miRNAs correspond to the mature forms of miR-141 (22bp) and miR-200c (23bp).

Given the pleiotropic role of miR-200c/141 cluster in tumorigenicity and invasiveness in cancer [2,4,14-17], we were interested to investigate the molecular mechanisms of its regulation. Previously published results pointed out the region encompassing positions -683 to -67 (relative to the precursor miRNA-200c (pre-miRNA-200c) first nucleotide) as relevant for transcription [18,19], therefore we concentrated our attention on this area. Sequence analysis revealed the presence of a well-defined CpG island between positions -343 to -115, upstream of the miR-200c/141 cluster (Fig. 1A). CpG islands (CpG dinucleotide-rich regions) are often co-localized with promoters as well as first exons of genes and methylation of the cytosines of the CpG dinucleotides frequently leads to transcriptional silencing [20]. In order to explore the possible relation between the detected CpG island and the transcripts containing the clustered miRNAs, 5' RACE experiments were performed using primers hybridizing specifically with the pre-miRNA-200c and pre-miR-141. For these experiments, was used RNA prepared from the breast cancer cell line MCF7 (epithelial-like and non-metastatic cell line derived from a pleural effusion of an invasive ductal breast carcinoma) which strongly expresses these two miRNAs [4,21]. The results show the existence of a prominent transcription starting site (TSS) located very close to the CpG island (position -93). This corresponds to a primary miRNA transcript incorporating both miRNAs as it was detected using both primers and it is located in a region that is highly conserved among mammals. A second TSS was identified within the CpG island, at position -285, and in close proximity to ZEB1 binding sites described by others [19] (Fig. 1). Interestingly, this TSS was not detected using the pre-miR-141 primer suggesting the existence of a transcriptional unit containing miR-200c, but not miR-141. Upon *in-silico *inspection, a high-score splice donor site (boundary exon/intron) was detected between the two miRNAs in position +373, supporting the existence of an alternative splicing variant that splices out miR-141 and conserves the miR-200c transcript. The relevance of this splice donor site for the generation of different splicing variants was not tested experimentally, but the data suggest that, although located in close proximity, these two miRNAs might not always be co-expressed (as suggested previously [22]). This might explain the observation previously made by us and others [4,23] that miR-200c is usually expressed at higher levels compared to miR-141.

In order to more closely characterize the miR-200c/141 promoter, luciferase reporter gene assays of the genomic region comprising the putative promoter region including both miRNAs (spanning the region between nucleotide -707 - +501 as defined in Fig. 1A) were performed. Indeed, strong promoter activity was detected in the respective region (Fig. 1B). Furthermore, to determine whether the putative RNA polymerase II (RNA Pol II) promoter is sufficient to enable proper downstream processing of both miRNAs, Northern blot analyses were performed. As shown in Fig. 1C, mature forms of both miRNAs were over-expressed in a time-dependent manner after transfection of Hela cells that express low levels of these two miRNAs.

In cancer, specific epigenetic changes are believed to be early events leading to subsequent changes in gene expression [13]. Given the reported role of miRNA-200c and miRNA-141 in metastasis formation [16,17] and, more recently, in tumorigenesis, development and stem cells homeostasis [14,24] we speculated that this locus might be subject to epigenetic regulation. To explore this, we used the MDA-MB-231 breast cancer cells (mesenchymal-like and highly metastatic cell line derived from a pleural effusion of an invasive ductal breast carcinoma) that under normal culture conditions express only residual amounts of these miRNAs [4,21]. We treated MDA-MB-231 cells with the DNA demethylating agent 5-AZA-CdR. The agent leads to irreversible inhibition of DNMT1, which is the maintenance DNA methyltransferase that copies methylation patterns to the newly synthesized DNA strand during DNA replication [25]. Notably, 5-AZA-CdR is a highly cytotoxic agent that in many cases leads to stalled cell proliferation and accelerated cell death during in vitro culture. In order to diminish these problems, in the present work we used mild dosages of 5-AZA-CdR (0,2 μM and 1 μM) (Additional file 1: Materials and Methods). This enabled successful propagation of cell culture experiments for periods of more than 30 days.

**Figure 2:**
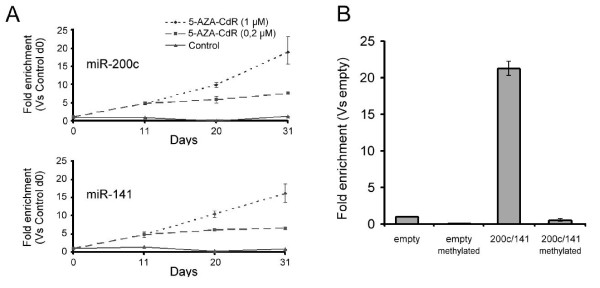
**The activity of the promoter is regulated by DNA methylation**. **(A) **TaqMan-based RT-PCR analysis of miR-200c and miR-141 in MDA-MB-231 kept under standard conditions and treated with 5-AZA-CdR at two different concentrations. Data are mean of triplicates and were normalized according to the ΔΔCt method using miR-16 as normalizer gene. **(B) **Firefly luciferase gene reporter assays of the miR-200c/141 promoter region in either demethylated or methylated states, respectively on Hela cells.

The treatment of MDA-MB-231 cells with 5-AZA-CdR led to the upregulation of both miRNAs (Fig. 2A) in a time and dosage-dependent manner. The expression of E-cadherin was previously shown to be regulated by DNA methylation [12,26], and as expected, treatment-dependent changes in miRNA expression were accompanied by transcriptional activation of E-cadherin in the formerly negative MDA-MB-231 line (Additional file 1: Supplementary Figure S1). Interestingly, we did not observe a decrease in expression of ZEB1 at mRNA or protein levels (data not shown), although it was described as a target of the miRNA-200 family [2-4]. As the action of 5-AZA-CdR is not selective, this result might indicate that ZEB1 is itself regulated by other mechanisms dependent on DNA methylation (e.g. third party transcription factors regulated by DNA methylation). Nonetheless, the above observations do not exclude that under physiological conditions, ZEB1 still is sensitive to changes in DNA methylation of the miR-200c/141 locus. Alternatively, the lack of ZEB1 downregulation could be due to the fact that the expression levels of miR-200c and -141 did not reach a certain threshold necessary to substantially reduce ZEB1 expression levels.

To exclude the possibility that activation of the miR-200c/141 cluster observed by chemical demethylation was mainly due to an indirect effect, e.g. activation of third party transcription factors, we next explored if expression of the miRNA cluster could be directly inhibited by DNA methylation. In order to reduce unspecific background signals we used an expression vector harboring a CpG-free luciferase transcriptional unit. This construct was methylated *in-vitro *using the DNA methylase Sss1 before being introduced into Hela cells. Indeed, after *in-vitro *methylation, promoter activity was strongly silenced (Fig. 2B) and together, these observations suggest a direct role of DNA methylation in transcriptional regulation of the miR-200c/141 cluster.

**Figure 3:**
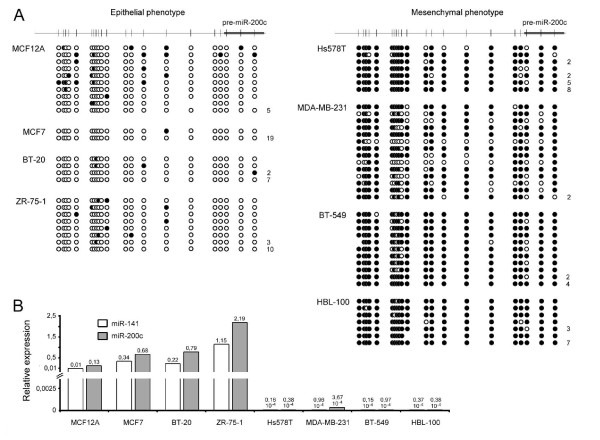
**Methylation status of the surrounding region correlates with miRNA expression and cellular phenotype**. **(A) **Methylation status analysis of the region surrounding miR-200c/141 in breast cancer cell lines of different phenotypes. White and black dots represent demethylated and methylated CpG dinucleotides, respectively. Each line represents an individual sequence and the number of identical clones presenting that sequence is indicated on the right. **(B) **Expression analysis of miR-200c and miR-141 in various breast cancer cell lines. Data are mean of triplicates and are calculated relative to miR-16 expression, which in all experiments did not exhibit significant differences in Ct values between samples.

Our observation, that the miR-200c/141 cluster is epigenetically regulated by DNA methylation, prompted us to investigate whether DNA methylation of these miRNAs correlates with their reported role in the regulation of tumorigenicity and invasiveness. For this purpose, we analyzed the DNA methylation status of the miR-200c/141 locus in a panel of 8 different breast cell lines of divergent origin. Some of these lines were originally established from breast cancer primary tumor cells (BT-20, BT-549 and Hs578T) while others were originated from breast cancer metastasis (MCF7, MDA-MB-231 and ZR-75-1) [21]. The HBL-100 cell line was established from an early lactation sample collected from an apparently healthy woman but during in-vitro culture evolved and became tumorigenic in nude mice [21]. The MCF12A cell line is a spontaneously immortalized cell line generated from normal breast epithelium [27]. Despite the different origins, the cell line panel can be grouped in terms of morphology and invasive capacity: MCF12A, MCF7, BT-20 and ZR-75-1 show an epithelial phenotype and no or low invasive capacity while MDA-MB-231, Hs578T, BT-549 and HBL-100 are clearly distinct showing a mesenchymal-like phenotype and high invasive capacity. Indeed, the degree of DNA methylation correlated strongly with the cellular phenotype (Fig. 3A): locus demethylation was consistently observed in four different breast cell lines with epithelial phenotype while strong DNA methylation was observed in the promoters of four cell lines representing mesenchymal phenotypes.

Consistent with previous observations [4], only breast cancer lines with an epithelial phenotype exhibited expression of the two miRNAs (Fig. 3B). Of note, the expression levels in the epithelial cell lines were substantially higher than the levels we could reach by demethylation of the mesenchymal MDA-MB-231 line, which is again consistent with incomplete demethylation of the miR-200c/141 promoter during 5-AZA-CdR treatment (Additional file 1: Supplementary Figure S2).

**Figure 4:**
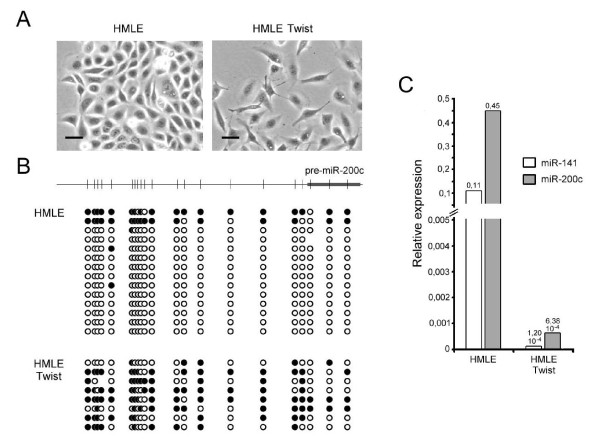
**Changes in DNA methylation of the miRNA locus occurring in an EMT *in-vitro *model**. **(A) **Phase contrast picture showing differences in cell morphology observed in epithelial HMLE breast cancer cells and in HMLE cells that underwent EMT by constitutively expressing Twist. *Scale bar*, 10 μm. **(B) **Methylation status analysis of the region surrounding miR-200c/141 in same cell populations shown in (A). **(C) **Expression analysis of miR-200c and miR-141 in same cell populations shown in (A). Data are mean of triplicates and are calculated relative to miR-16 expression, which did not exhibit significant differences in Ct values between samples.

To further explore the role of DNA methylation for repression of the miR-200c/141 cluster in the course of EMT, we took advantage of an *in-vitro *EMT model, which is based on ectopic expression of the EMT transcription factor Twist in non-transformed immortalized human mammary epithelial cells (HMLE) [28]. Expression of Twist (HMLE-Twist) reproducibly led to loss of epithelial cell-cell adhesion and acquisition of mesenchymal morphology as well as to induction of ZEB1 and ZEB2 expression and downregulation of E-cadherin (Fig. 4A and Additional file 1: Supplementary Figure S3). The EMT process was accompanied by DNA hypermethylation and transcriptional silencing of the miR-200c/141 promoter (Fig. 4B and 4C). Importantly, although DNA methylation levels of Twist-transfected HMLE cells (56,0% counting all CpG dinucleotides from all clones) were not as high as in invasive cell lines of mesenchymal phenotype (Fig. 3A) (on average 89,3%), they were nonetheless associated with strong downregulation of the respective miRNAs (Fig. 4B and 4C). The data thus suggest that effective silencing of the miR-200c/141 locus is already achieved by intermediate levels of DNA methylation.

During preparation of this manuscript, a correlation between the expression levels of miR-200c/141 and the degree of DNA methylation of the promoter-associated CpG island was also reported by Vrba and colleagues [29]. Similar to the present study, the authors demonstrate a correlation of DNA methylation levels with invasive phenotype and the capacity of 5-AZA-CdR to reactivate the expression of the formerly silenced miRNAs in invasive breast cancer cell lines. The present study goes beyond the correlative analysis by providing evidence that expression of the miR-200c/141 locus is indeed partly controlled by DNA methylation. Firstly, in vitro methylation experiments showing how DNA methylation of the miR200c/141 locus shuts down expression of miR-200c and miR-141 provides a functional link between DNA methylation of the promoter and expression of the miR-200c/141 locus. Secondly, ectopic expression of the EMT inducer Twist led to a limited increase of DNA methylation in the miR200c/141 promoter, which was nevertheless accompanied by complete shut down of miRNA expression. The latter data indicate that even limited levels of DNA methylation can cause transcriptional silencing of the miRNA locus. Importantly, the effect of Twist on DNA methylation levels shown in our study further stresses the functional relevance of epigenetic changes in the miR200c/141 locus and suggests a potential role for epigenetic regulation of EMT.

Although the present work supports the idea that changes in DNA methylation of this particular locus might be involved in EMT, it remains to be determined if the initial trigger to shutdown the miR-200c/141 promoter during the EMT process is given by changes in DNA methylation levels or binding of repressors (as ZEB1, ZEB2, or Twist) to the promoter or if several of these repressor mechanisms act simultaneously and synergistically. In the latter case, DNA methylation of miRNAs in conjunction with ZEB1 expression would then support transition to a mesenchymal phenotype. Interestingly, in clones established after experimental knockdown of ZEB1 in MDA-MB-231 cells, others observed an upregulation of miR-200c/141 expression [19]. This opens the possibility that ZEB1 might be necessary for maintaining DNA methylation of the miR-200c/141 promoter. In this regard, it is known that ZEB1 interacts with CtBP [30], that in turn interacts with components of the Polycomb complex [31]. As these complexes promote DNA methylation via interaction with DNMTs [32], ZEB1 could indeed enforce DNA methylation of the miR-200c/141 promoter. These questions surely deserve further investigation.

## Competing interests

The authors declare that they have no competing interests.

## Authors' contributions

RN designed and conducted the main experiments, analyzed the data and wrote the manuscript. CS established Twist transfectants and contributed to the design of the study. EH and DN provided breast cancer cell lines and provided confirmatory data. KMI and H-IT performed Northern blots. SW performed microRNA multiplex analysis. SW, H-IT, and PW critically revised the manuscript. MU and SS supported financially the project and contributed to the design of the study, data interpretation and writing of the manuscript. All authors read and approved the final manuscript.

## Supplementary Material

Additional file 1 **Supplementary figures, materials and methods and sequences of primers and probes**. This file contains 3 figures followed by the respective legends. These figures are referred on the main text of the manuscript as 'Additional file 1: Supplementary Figure S1, S2 and S3'. Additionally, the document contains also the description of methods used and the sequences of primers and probes used on real-time-PCR experiments, analysis of methylation status, cloning of promoter, 5'RACE experiments and Northern blots. Click here for file
