# Supplementary material for: Role of DNA methylation in miR-200c/141 cluster silencing in invasive breast cancer cells
Source: BMC Res Notes. 2010 Aug 3;3:219. doi: 10.1186/1756-0500-3-219 (PMC3161370; doi:10.1186/1756-0500-3-219)
Supplement: Additional file 1 — Supplementary figures, materials and methods and sequences of primers and probes. This file contains 3 figures followed by the respective legends. These figures are referred on the main text of the manuscript as 'Additional file 1: Supplementary Figure S1, S2 and S3'. Additionally, the document contains also the description of methods used and the sequences of primers and probes used on real-time-PCR experiments, analysis of methylation status, cloning of promoter, 5'RACE experiments and Northern blots. [file 1756-0500-3-219-S1.PDF]

Additional File 1

Additional File 1 title: Supplementary figures, materials and methods and sequences of primers and probes

Additional file 1 description: This file contains 3 figures followed by the respective legends. These figures are referred on the main text of the manuscript as 'Additional file 1: Supplementary Figure 1, 2 and 3'. Additionally, the document contains also the description of methods used and the sequences of primers and probes used on real-time-PCR experiments, analysis of methylation status, cloning of promoter, 5'RACE experiments and Northern blots.

Supplementary Figure 1

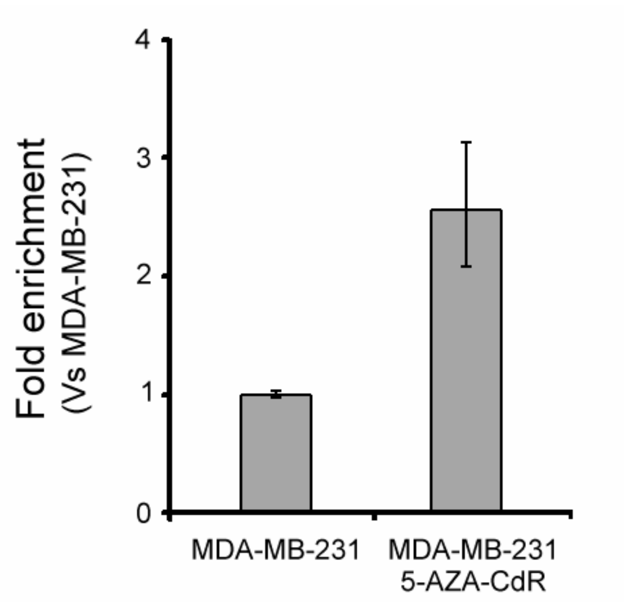

Supplementary Figure 1 - Upregulation of E-Cadherin on MDA-MB-231 cells after treatment with 0,2µM of the DNA demethylating agent 5-AZA-CdR for 18 days. Expression was detected by SYBR Green-based real-time PCR and normalized using the ΔΔCt method and β-actin expression levels detected by the same method. Data are mean of triplicates.

Supplementary Figure 2

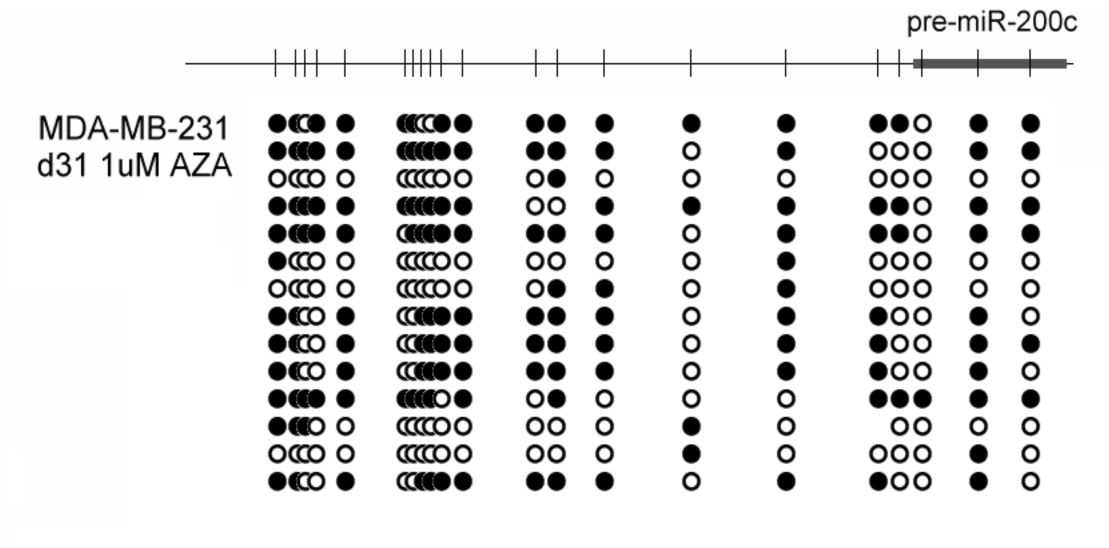

Supplementary Figure 2 - Methylation status analysis of the region surrounding miR-200c/141 in MDA-MB-231 cells after treatment with 0,2µM of the DNA demethylating agent 5-AZA-CdR for 18 days. The analysis was performed as described in Materials and Methods. Open and closed dots represent demethylated and methylated CpG dinucleotides, respectively. Each line represents an individual cloned sequence. The methylation level and patterns differ from the ones obtained for control MDA-MB-231 cells (Fig. 3A). Considering the 21 CpGs dinucleotides analyzed, a decrease of 20,5% is observed in the methylation level following 5-AZA-CdR treatment (from 71,7% in control MDA-MB-231 cells (Fig. 3A) to 51,2% in 5-AZA-CdR-treated MDA-MB-231 cells).

Supplementary Figure 3

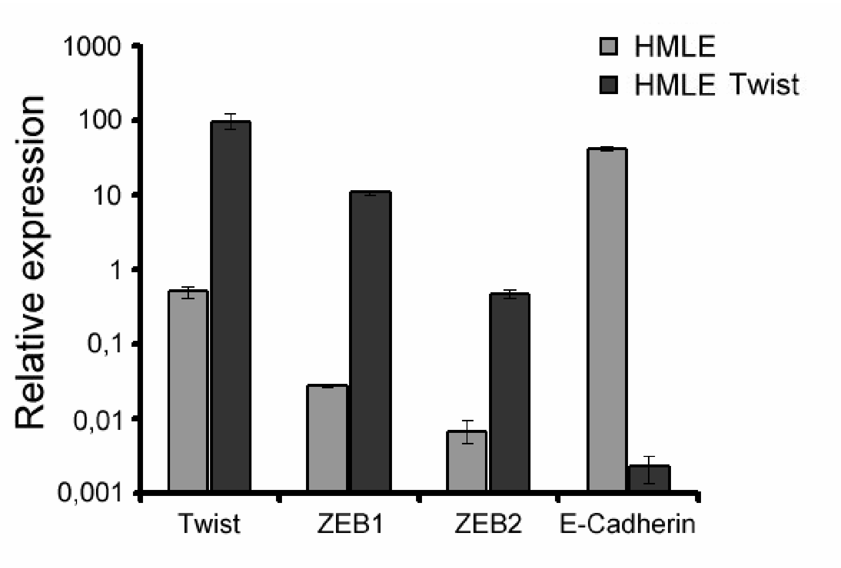

Supplementary Figure 3 – Expression levels of Twist, ZEB1, ZEB2 and E-cadherin on HMLE cells and on HMLE cells ectopically expressing Twist. Total RNA was extracted using the Qiagen Mini RNA extraction kit. 500ng-2ug of RNA were used for first strand synthesis using the Superscript III kit (Invitrogen). SYBR Green Mix I (Roche Diagnostics) was used for amplification and samples were run in triplicates on a Lightcycler Instrument (Roche Diagnostics). All quantifications were normalized to an endogenous control L32. The relative quantification value for each target gene is expressed as $2^{-(Ct-Cc)}$ (Ct and Cc are the mean threshold cycle differences of target gene and L32 control, respectively). The relative expression levels of samples are presented by a semi-log plot.

Material and Methods

Cell culture, transfection. All cell lines used on this work were maintained under standard culturing conditions. Treatment of MDA-MB-231 cells with 5-aza-2´-deoxycytidine (5-AZA-CdR) was performed maintaining a 0,2µM concentration of the demethylating drug for 31 days on the standard culturing medium. At day 11 of culture with AZA, the culture was split and a fraction was from then on incubated in the presence of 1µM of the same drug. aHela cells (cultured on DMEM; 10% FCS) and MCF7 cells (cultured on RPMI; 10% FCS) were transfected using 2µg of plasmid DNA and FUGENE® HD Transfection Reagent (Roche) according to the manufacturer's instructions. Immortalized human mammary epithelial cells (HMLE) were generated through introduction of hTERT, partially transformed by introduction of the SV40 large T antigen (HMLE) and maintained as previously described [Supplementary Reference 1]. HMLE-Twist cells were derived by transduction with the pBABE-puro-Twist retroviral expression vector containing the cDNA for mouse Twist as described previously [Supplementary Reference 2].

RNA preparation, RT-PCR, Northern blot. Total RNA for Northern blot and RT-PCR was extracted using Trizol® reagent (Invitrogen). For Northern blot, 7µg of total RNA extracted using Trizol® reagent (Invitrogen) was separated on a standard denaturing 15% (w/v) polyacrylamide gel and blotted unto a neutral nylon membrane (Amersham biosciences, U.S.A.). Membrane cross link was performed using 1-ethyl-3-(3-dimethylaminopropyl) carbodiimide (EDC) (Sigma, U.S.A.) as previously described [Supplementary Reference 3]. Specific detection of RNA U6, miR-200c and miR-141 was performed using probes containing LNA modified based and radioactively labelled. For semi-quantitative RT-PCR, 1µg of RNA was converted into cDNA using M-MLV RT (Promega) and oligo(dT) primers according to manufacturer's instructions. Real-time PCR was performed using QuantiTect® SYBR® Green PCR (Qiagen) on an ABI Prism 7700 Sequence detector (Perkin Elmer). Normalization of expression levels was done by the ΔΔCt method relative to β-actin. All primers and probes used are listed on "Additional file 1: Primers and Probes".

miRNA expression profiling. The expression of miR-141 and miR-200c in different breast cancer cell lines was measured using Individual TaqMan® MicroRNA Assays (ABI)

[Supplementary Reference 4] according to the manufacturer's instructions. The data were normalized to miR-16 expression measured using the same method.

DNA preparation and DNA methylation analysis. DNA was extracted using QIAamp® DNA Blood Mini Kit (Qiagen). Bisulfite conversion was performed using EpiTect® Bisulfite Kit (Qiagen) according to the manufacturer's instructions and using 1µg of DNA. DNA methylation patterns were analyzed by genomic sequencing of bisulfite-converted DNA as described previously [Supplementary Reference 5]. All primers used are listed on "Additional file 1: Primers and Probes".

Gene reporter assays. To check for promoter activity of the region upstream of the miR141/200c cluster, the defined region was amplified by PCR from genomic DNA and cloned into pMOD vector (Invivogen). All primers used are listed on "Additional file 1: Primers and Probes". In vitro DNA methylation of pMod vectors containing various inserts upstream the Luciferase gene, was performed using the CpG methyltransferase SssI (NEB). Cells were harvested 24h after transfection and firefly luciferase activity was measured using Luciferase Reporter Gene Assay Kit (Roche). Data were normalized using protein content measured with DC Protein Assay kit (Biorad) following manufacturer's instructions.

5' Rapid Amplification of cDNA ends (5' RACE). 5' RACE experiments were performed using 5'/3' RACE Kit, 2nd Generation (Roche) according to manufacturer's protocols using 1µg of total RNA extracted from MCF7 cells. PCR products were cloned using TA cloning Kit (Invitrogen) and individually sequenced. All primers used are listed on "Additional file 1: Primers and Probes".

Primers and Probes

Primers used for transcript detection/quantification in real-time-PCR experiments:

E-cadherin for, 5'-AAGAAGGAGGCGGAGAAG
E-cadherin rev, 5'-CATAGTCAAACACGAGCAGAG

β-actin for, 5'-GAAGATCCTCACCGAGCGC
β-actin rev, 5'-AGGGTAGATGGTGGTGCCG

Primers used for methylation status analysis of the miR-200c/141 locus:

BSC-200c/141 for, 5'-TTGAGTTTGGGATTGTAG
BSC-200c/141 rev, 5'-AAAACCTCCATCATTACC

Primers used to clone the surrounding region/putative promoter region of miR-200c/141:

200c/141 for, 5'-GAAGATCTTCCCGCTTTTTGTACCTCTGGAG
200c/141 rev, 5'-CCCAAGCTTGGGCTGCCGAGAGAACCCACC

(-) control for, 5'-GAAGATCTTCAGAGCCTTGCCGTAACAGA (6th intron of KIR3DL2 gene)
(-) control rev, 5'-CCCAAGCTTGGGCCCTCCCATTTAACCATC

Primers used in 5'RACE experiments:

200c SP1, 5'-CTCCATCATTACCCGGCAGT
200c SP2, 5'-ATTAGAGACTCCCAACCGCA
200c SP3, 5'- AAACACTGCTGGGTAAGACGA

141 SP1, 5'-GGGAGCCATCTTTACCAGAC
141 SP2, 5'-CCAGACAGTGTTAGGAGCTTCA
141 SP3, 5'-ACTGTACTGGAAGATGGACCC

200c+141 Oligo-dT-anchor primer, 5'- GACCACGCGTATCGATGTCGAC(16T)V
200c+141 anchor primer, 5'-GACCACGCGTATCGATGTCGAC

Probes used for Northern blots:

miR-141, 5'- CCA*TCT*TTA*CCA*GAC*AGT*GTT*A

miR-200c, 5’- TCC*ATC*ATT*ACC*CGG*CAG*TAT*TA
U6 RNA, 5’- GCC*ATG*CTA*ATC*TTC*TCT*GTA*T

LNA modified bases are signed with a * after the respective base letter.

Supplementary Reference List

1. Elenbaas B, Spirio L, Koerner F, Fleming MD, Zimonjic DB, Donaher JL, Popescu NC, Hahn WC, Weinberg RA: **Human breast cancer cells generated by oncogenic transformation of primary mammary epithelial cells.** *Genes Dev* 2001, **15:**50-65.

2. Yang J, Mani SA, Donaher JL, Ramaswamy S, Itzykson RA, Come C, Savagner P, Gitelman I, Richardson A, Weinberg RA: **Twist, a master regulator of morphogenesis, plays an essential role in tumor metastasis.** *Cell* 2004, **117:**927-939.

3. Pall GS, Codony-Servat C, Byrne J, Ritchie L, Hamilton A: **Carbodiimide-mediated cross-linking of RNA to nylon membranes improves the detection of siRNA, miRNA and piRNA by northern blot.** *Nucl Acids Res* 2007, **35:**e60.

4. Chen C, Ridzon DA, Broomer AJ, Zhou Z, Lee DH, Nguyen JT, Barbisin M, Xu NL, Mahuvakar VR, Andersen MR et al.: **Real-time quantification of microRNAs by stem-loop RT-PCR.** *Nucleic Acids Res* 2005, **33:**e179.

5. Daskalakis M, Nguyen TT, Nguyen C, Guldberg P, Kohler G, Wijermans P, Jones PA, Lubbert M: **Demethylation of a hypermethylated P15/INK4B gene in patients with myelodysplastic syndrome by 5-Aza-2'-deoxycytidine (decitabine) treatment.** *Blood* 2002, **100:**2957-2964.
